# Thermodynamic, chemical, and electrochemical studies of Aloe ferox Mill extract as a naturally developing copper corrosion inhibitor in HCl solution

**DOI:** 10.1038/s41598-024-62169-x

**Published:** 2024-05-24

**Authors:** Kaseb D. Alanazi, Basmah H. Alshammari, Tahani Y. A. Alanazi, Odeh A. O. Alshammari, Ashraf M. Ashmawy, Meshari M. Aljohani, Isma Haq, Reda Abdel Hameed, M. A. Deyab

**Affiliations:** 1 Department of Chemistry, College of Science, University of Ha’il, 81442 Hail, Saudi Arabia; 2 Chemistry Department, Faculty of Science (boys), Al-Azhar University, Cairo, 11884 Egypt; 3 Department of Chemistry, College of Science, Tabuk University, Tabuk, Saudi Arabia; 4 Institute of Chemistry, University of the Punjab, Quaid-e-Azam Campus, Lahore, Pakistan; 5 Basic Science Department, Preparatory Year, University of Ha’il, 1560 Hail, Kingdom of Saudi Arabia; 6 Egyptian Petroleum Research Institute (EPRI), Nasr City, Cairo Egypt

**Keywords:** Copper, Cleaning process, Corrosion inhibitors, Extract, Desalination, Chemistry, Electrochemistry

## Abstract

Copper can be susceptible to corrosion in acidic cleaning solutions for desalination system, especially if the solution is highly concentrated or if the cleaning process involves extended exposure to the acid. In the current work, Aloe ferox Mill (AFM extract) can be used as a natural origin corrosion inhibitor for copper in 1.0 M HCl solution. The corrosion mitigation qualities of AFM extract were assessed by means of electrochemical, gravimetric, and surface examinations. AFM extract is a mixed-type inhibitor, based on polarization research findings. The inhibitory effectiveness of AFM extract rises with concentration, reaching its maximum level (93.3%) at 250 mg L^–1^. The inclusion of AFM extract raises the activation energy for the corrosion reaction from 7.15 kJ mol^–1^ (blank solution) to 28.6 kJ mol^–1^ (at 250 mg L^–1^ AFM extract).

## Introduction

Copper is commonly used for piping and tubing in desalination plants due to its excellent corrosion resistance and compatibility with seawater. Copper pipes are often utilized for conveying seawater, brine, and other fluids within the desalination system. Copper’s corrosion resistance helps ensure the longevity and reliability of the piping infrastructure. Copper is reactive to acids, and when exposed to acidic cleaning solutions, it can undergo a chemical reaction known as acid attack^1,2^. Acids can react with the copper surface, dissolving the metal and forming copper salts or complexes. This process leads to the corrosion and degradation of the copper material^3^. Different types of acids can have varying corrosive effects on copper. Acids, such as citric acid or hydrochloric acid can be utilized for cleaning and maintenance purposes in desalination systems^4,5^. However, it's crucial to follow manufacturer guidelines and industry best practices to ensure safe and effective cleaning procedures.

Organic acids, such as acetic acid (found in vinegar) or citric acid, are generally less corrosive to copper, but prolonged exposure or high concentrations can still lead to corrosion.

Many plants contain compounds with corrosion inhibiting properties, such as tannins, flavonoids, alkaloids, and phenolic compounds^6–9^. Examples include extracts from plants like Aloe vera, green tea, grape seed, pomegranate, neem, and rosemary. These extracts have been studied for their ability to form protective films on metal surfaces and inhibit corrosion^10,11^.

It's important to note that the use of natural extracts as corrosion inhibitors may have limitations in terms of stability, consistency, and long-term effectiveness compared to synthetic inhibitors. Therefore, thorough testing and evaluation of the extracted material's performance, as well as compatibility with the copper substrate and the specific environment, are crucial before considering its acid cleaning application. We examined the anticorrosion properties of AFM extract using chemical and electrochemical studies.

Using AFM extract as corrosion inhibitor for copper in HCl acid is an interesting and potentially novel research area. Conduct in-depth mechanistic studies to understand the adsorption behavior and protective mechanisms of the extract corrosion inhibitors on the copper surface. Utilize advanced surface analysis techniques, such as electrochemical, gravimetric, and surface examinations, to gain insights into the adsorption kinetics, film formation, and barrier properties of the extract inhibitors. By exploring these novel aspects, we can contribute to the advancement of knowledge in the field of using plant extracts as corrosion inhibitors for copper in HCl acid.

The usage of AFM extract as a corrosion inhibitor has various benefits.

AFM extract is abundant and widely available, making it a cost-effective option for industrial applications. Furthermore, the extract is derived from renewable resources. This assures a steady supply of corrosion inhibitors while minimizing environmental effect. From a cost standpoint, these compounds can produce protective covers on metal surfaces, preventing corrosion even at low concentrations. They are also often straightforward to work with and may be combined into a variety of corrosion inhibitor compositions, such as solutions, suspensions, and coatings.

## Experimental part

### Materials

In this experiment, copper samples with composition (%wt) copper 98%, Phosphorus 0.04%, Carbon 0.005, Silicon 0.2, Fe 0.18% and Zn 1.57% were evaluated. Before evaluation, the copper specimen was washed with a solution of ethyl alcohol and filtered water, and it was sanded with emery sheets varying within grade between 600 and 1200. 1.0 M HCl solutions have been generated for each research employing water that had been de-ionized and analar class HCl (Merck & Co). AFM extract (Aloe ferox Mill) was obtained from Egyptian company.

Soxhlet extraction method was used involves continuous extraction using an ethanol (purity 99%) solvent, at elevated temperatures. The AFM extract was totally soluble in both water and hydrochloric acid.

### Electrochemical and chemical experiments

For the electrochemical tests, a potentiostat/3000-Gamry was being used. The working component of the tests was a copper plate with a functional area of 0.469 cm^2^, the counter component was a Pt strip, and the reference component was a saturated calomel electrode (SCE). The experiments were carried out in a double-walled cell. Tafel polarisation graphs were created by applying varying voltage ranging (± 0.250 V/SCE vs. OCP) to the copper anode using a scan rate of 1.0 mV s^–1^.

 Experiments using electrochemical impedance spectroscopy (EIS) were conducted at frequencies between 0.01 Hz and 100 kHz at an open circuit potential using voltage amplitude of 10 mV.

The electrochemical tests were carried out repeatedly to ensure their accuracy.

Copper sheets were divided into pieces sized 1.8 cm × 1.0 cm × 0.05 cm in order to assess gravimetric tests. The solutions being examined (100 ml) were immersed in the copper plates. The standard procedure G1-03–2017-e1 ASTM was utilized to determine the gravimetric^12^. Each of the samples was immersed in liquids for a whole day. Three replicates of the experiments were conducted, and the average loss of mass was ascertained.

The following equation was used for calculating the copper (*C*_R_) corrosion rate^13^:

1 $$C_{R} = \frac{W}{A \times t}.$$

(*C*_R_ = corrosion rate, *W* = mass loss (mg), *A* = cu surface area (cm^2^), *t* = time (h)).

Objectives were made to do many tests using a water bath with temperature controls at different temperatures (298, 313, 323, and 333 K).

### Surface investigations

The copper surface was analyzed using a range of surface characterization techniques. Throughout the tests, the electrodes were immersed in 1.0 M HCl solution incorporating AFM extract (250 mg L^-1^) for a period of three hours. They were washed with water that was distilled and dried out after being removed from their cells. The surface of copper was examined using scanning electron microscopy (SEM) and energy dispersive X-ray (EDAX) (system ZEISS EVO). FTIR analysis was performed on both the pure AFM extract and the corrosion products using Agilent FTIR device.

## Results and discussion

### Electrochemical studies

**Figure 1:**
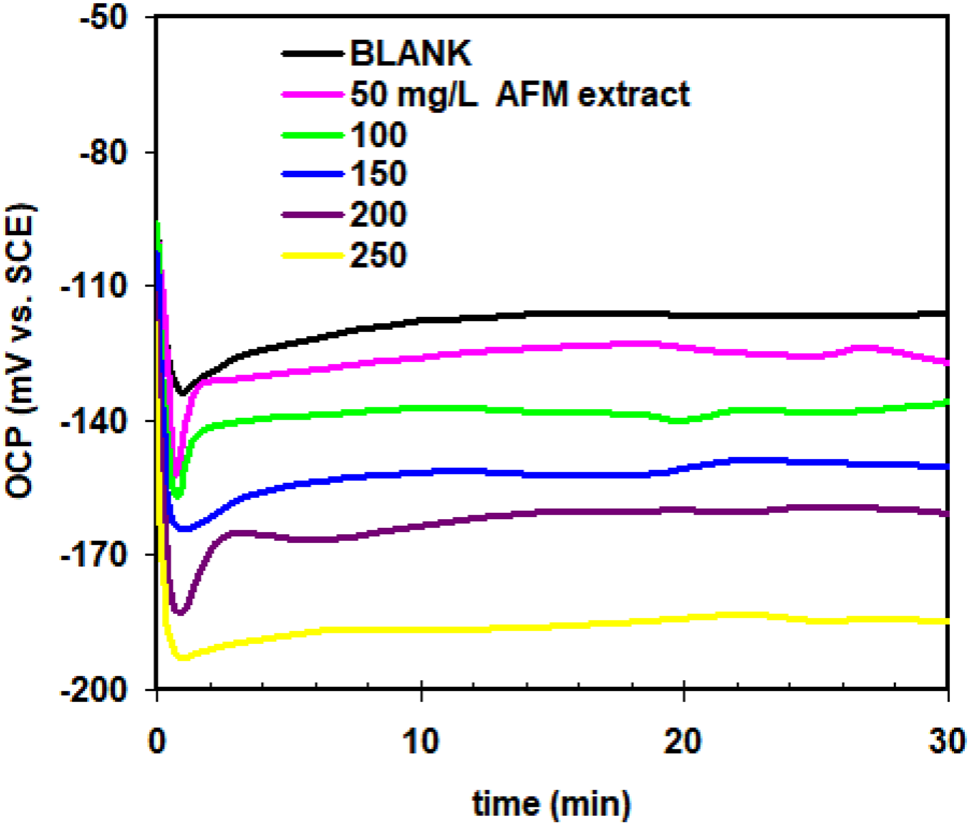
Open circuit potential curves for copper in 1.0 M HCl without and with different AFM extract concentrations added at 298 K.

For 30 min, the open circuit potential (OCP) measurements for copper in 1.0 M HCl were identified both with and without the incorporation of AFM extract. The resulting curves are displayed in Fig. 1. Compared to the trends when AFM extract is present, the OCP in the blank solution is less dramatically altered to more negative values. This actually to cause by of corrosion products adhering to the copper surface. The examination of the OCP values in uncontrolled and inhibited liquids at the end of the tests allowed for the classification of the AFM extract as a mixed-type inhibitor, which has a stronger impact on the cathodic operation^14^.

**Figure 2:**
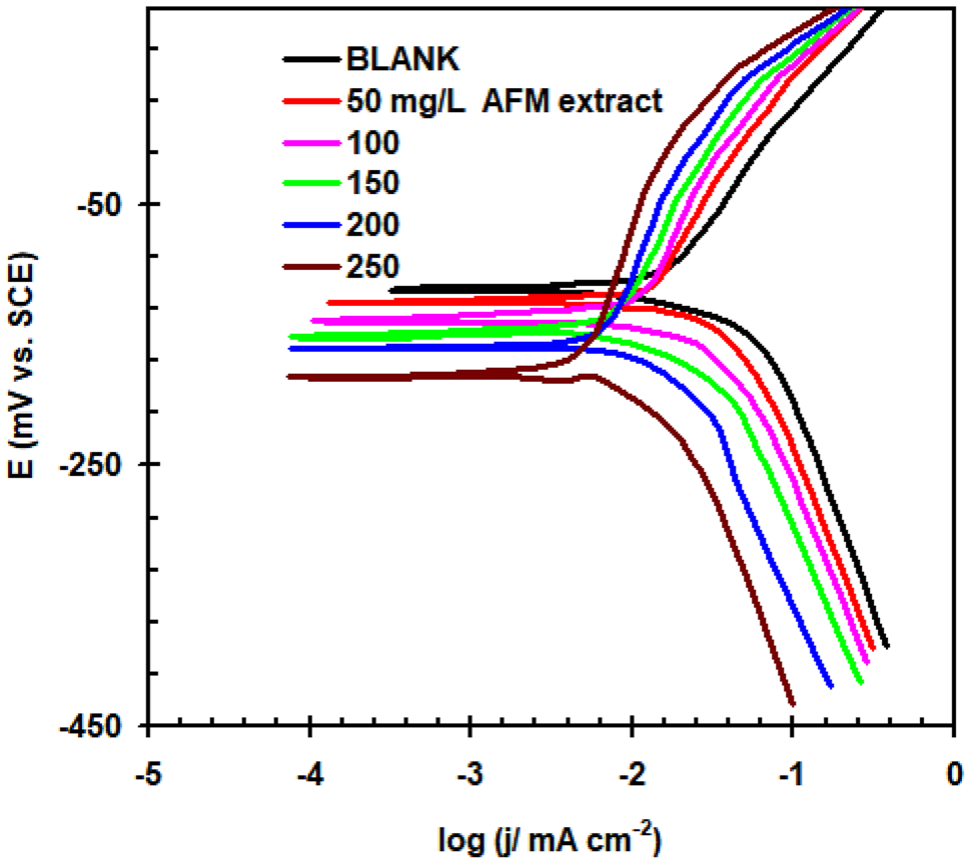
Tafel polarisation plot of copper in 1.0 M HCl without and with different AFM extract concentrations added at 298 K.

Figure 2 shows the Tafel polarization plot regarding copperin 1.0 M HCl with various dosages of AFM extract at 298 K. The polarization analysis demonstrated that each of the cathodic and anodic currents is changed when the amount of AFM extract is increased. Table 1 gives the Tafel polarization results. There is not a noticeable trend in the corrosion potential (*E*_corr_) measurements. The AFM extract's mixed type behavior was supported by the difference in *E*_corr_ values between the acid solutions including the extract and the blank acid solution, which showed to indicate below 85 mV^14^.

**Table 1:** Polarization parameters for copper in 1.0 M HCl solution in the absence and presence of AFM extract at 298 K.

AFM extract (mg L^–1^)	* − E*_corr._ mV (SCE)	*j*_corr._ μA cm^–2^	β_a_ (mV dec^−1^)	* − *β_c_ (mV dec^−1^)	*η*_j_%
Blank	114 ± 2.6	28.7 ± 1.2	67	178	–
50	125 ± 2.7	20.5 ± 1.1	66	164	28.5
100	139 ± 3.1	11.4 ± 0.9	73	156	60.2
150	151 ± 3.2	6.3 ± 0.6	78	177	78.0
200	160 ± 3.8	4.6 ± 0.3	83	152	83.9
250	182 ± 3.6	1.9 ± 0.2	88	134	93.3

It is evident that the anodic and cathodic processes' mechanisms were unaffected by the addition of AFM extract to the corrosion surroundings, since no significant modifications were seen in the cathodic Tafel slop (β_c_) or anodic Tafel slop (β_a_)^15–17^.

The corrosion current density (*j*_corr_) results show a considerable drop with the addition of AFM extract. This suggests that in 1.0 M HCl solutions, AFM extract inhibits copper anode corrosion^18^. Using the following formula, the AFM extract's inhibitory ability (η_j_%) was calculated^19^:

2 $$\eta_{j} \% = \frac{{j_{corr(0)} - j_{corr} }}{{j_{corr(0)} }} \times 100,$$

(*j*_corr(0) =_ corrosion current density in blank solution, *j*_corr =_ corrosion current density in the presence of AFM extract).

The inhibitory capacity of AFM extract increases with concentration, reaching its highest value (93.3%) at 250 mg L^–1^. These results support the significant inhibitory action of AFM extract regarding copper corrosion in 1.0 M HCl.

**Figure 3:**
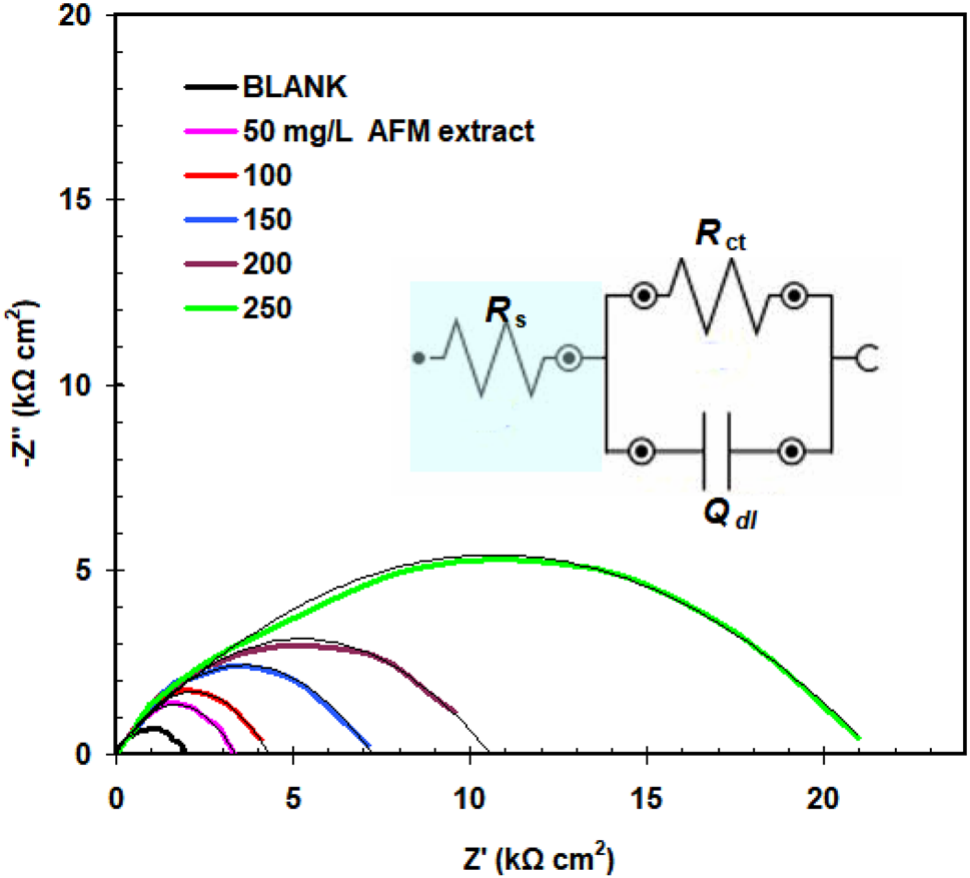
Nyquist curves of copper in 1.0 M HCl without and with different AFM extract concentrations added at 298 K (inset: equivalent circuit used to fit the EIS data).

EIS tests were conducted to more thoroughly examine the impact of AFM extract on the corrosion behavior of copper in 1.0 M HCl. Figure 3 displays the acquired results (Nyquist diagram). Table 2 summarizes the EIS parameters that were determined by fitting, as indicated by Nyquist diagram. It is evident from examining the Nyquist diagram (Fig. 3) that the semicircle width raises with increasing AFM extract concentration. Corrosion rate is lowered as a result.

**Table 2:** EIS parameters for copper in 1.0 M HCl solution in the absence and presence of AFM extract at 298 K.

AFM extract (mg L^–1^)	*R*_ct_ kΩ cm^2^	*Q*_dl_ μF cm^–2^	χ^2^	*n*	*η*_R_%
Blank	1.90 ± 0.15	622 ± 3.4	1.8 × 10^−3^	0.76	–
50	2.80 ± 0.19	502 ± 2.9	1.7 × 10^−3^	0.80	32.1
100	3.98 ± 0.21	442 ± 2.5	1.6 × 10^−3^	0.82	2.52
150	7.14 ± 0.22	317 ± 3.3	1.1 × 10^−3^	0.89	3.73
200	9.90 ± 0.25	234 ± 3.9	1.3 × 10^−3^	0.92	8.80
250	22.3 ± 0.26	101 ± 2.0	1.6 × 10^−3^	0.92	91.4

The inset of Fig. 3 illustrates the proposed equivalent circuit that was used to analyze and simulate the obtained graphs. The charge transfer resistance is represented by *R*_ct_, the solution resistance by *R*_s_, and the ideal capacitor is replaced by the constant phase element (Q_dl_)^20^. The accuracy of the fitting procedures is shown by the relatively low goodness of fit (χ2) results in Table 2. The data displayed in Table 2 demonstrate that when AFM extract is present, "n" values rise, indicating an improvement in surface uniformity as a result of AFM extract adsorption.

Furthermore, as the concentration of AFM extract increased, values of Q_dl_ decreased. This is related with the adsorption of AFM extract molecules on the copper surface leading to decrease exposed copper surface to aggressive ions. The following equation was utilized to calculate the inhibitory efficacy (*η*_R_%).

3 $$\eta_{R} \% = \frac{{R_{ct} - R_{cto} }}{{R_{ct} }} \times 100,$$

(*R*_cto_ is recoded in 1.0 M HCl solution and *R*_ct_ is recoded in the presence of AFM extract).

Table 2 lists the calculated inhibition efficiencies. The results derived by polarization (Table 1) agree with the computed EIS values (Table 2).

The adsorption of AFM extract on the copper surface can indeed play a crucial role in inhibiting corrosion in HCl solution^21–23^. The adsorption can occur through various mechanisms, such as chemisorption or physical adsorption. Chemisorption involves a strong chemical interaction between the inhibitor and the metal surface, while physical adsorption involves weaker, non-specific interactions. When the AFM extract adsorbs onto the copper surface, it forms a protective barrier that acts as a physical or chemical shield against corrosive species. This barrier can inhibit the diffusion of corrosive ions or molecules, reducing their access to the copper surface. The effectiveness of corrosion inhibition is often related to the surface coverage of the AFM extract molecules on the copper surface. Higher surface coverage leads to greater protection by forming a denser and more uniform layer, preventing or reducing the contact between the corrosive environment and the copper surface.

### Chemical studies

As can be observed from the results shown in Table 2, the method of gravimetric measurements was used to assess the weight loss rate and extent of protection for copper in 1.0 M HCl solution in both the presence and the absence of AFM extract at 298 K. The equation below was utilized to quantify the *η*_W_% inhibitory effectiveness of AFM extract^24^:

4 $$\eta_{W} \% = \frac{{C_{R0} - C_{R} }}{{C_{R0} }} \times 100.$$

(*C*_R0 =_ corrosion rate in blank solution, *C*_R=_ the corrosion rate in the presence of AFM extract).

The *C*_R_ and *η*_W_% at varying quantities of AFM extract are outlined in Table 3. As the concentration of AFM extract rises, the value of *C*_R_ keeps reducing. This decrease in *C*_R_ as AFM extract concentration rises is indicative of a tendency towards increased surface coverage of the copper via AFM extract molecules. The maximum inhibitory efficiency (*η*_W_% = 91.0) was seen at a 250 mg L^–1^ concentration of AFM extract.

**Table 3:** Gravimetric parameters of copper at 298 K in 1.0 M HCl solution with and without AFM extract.

AFM extract (mg L^–1^)	*C*_R_	*η*_*W*_%
	(μg cm^–2^ h^–1^)	
Blank	54.9 ± 2.4	–
50	41.0 ± 2.3	25.3
100	25.5 ± 1.8	53.5
150	15.0 ± 1.1	72.6
200	10.5 ± 0.5	80.8
250	4.9 ± 0.4	91.0

Many studies investigated the inhibitory effects of various extracts on copper corrosion inside HCl solution. We conducted a comparison of our findings with earlier research published on copper corrosion inhibition in acidic conditions. Wu et al.^25^ investigated the corrosion inhibitory effects of psidium guajava linn leaves extract on copper in a sulfuric acid solution. Once the amount of psidium guajava linn leaves extract achieves 600 mg/L, it may suppress corrosion by 95%. Kathiravan et al.^26^ used electrochemical methods to study the protection of Ruellia Tuberosa L leaves extract against copper in 0.5 M HCl. The extract at a concentration of 0.18 g/L provided 87.01% protection. Ahmed and Zhang^27^ have evaluated the inhibitory action of Atriplex leucoclada extract by experimental investigations for copper in1 M HCl. At a concentration of 8 g/L, Atriplex leucoclada extract had the highest inhibitory performance (91.5%). Jmiai et al.^28^ studied the the protection of Jujube shell extract in 1 M HCl utilizing chemical and electrochemical strategies. The findings showed a maximal inhibitory effectiveness of 91.0% at 1 g/L. Compared to the majority of the values given in the published research, AFM extract has a distinct superiority in considerations of both dose and matching efficiency, showing that AFM extract is an efficient corrosion inhibitor, and hence its use in copper protection is critical.

### Thermodynamic studies

Thermodynamic studies provide valuable insights into the fundamental mechanisms of corrosion inhibition of AFM extract^29–31^.

In 1.0 M HCl solution, both with and without AFM extract (250 mg L^–1^), Table 4 shows the *C*_R_ and *η*_W_% for copper as dependent variables of temperature (298 to 333 K). Evidence from the data indicates that when the temperature goes up, the *C*_R_ of copper in acid solution, whether blank or controlled tends increases. A change in the adsorption/desorption equilibrium towards the de-sorption of AFM extract from the copper surface as well as the roughness of the copper surface can explain this tendency^32^. As the temperature goes up, the *η*_W_% continuously decreases (Table 4), suggesting a physisorption mechanism^33^. The fact that increasing temperatures have minimal impact on *η*_W_% indicates that the AFM extract can be seen of as an efficient inhibitor, especially at high temperatures. Thermodynamic parameters, such as enthalpy (∆H*) and entropy (∆S*), can be determined to assess the nature and mechanism of the corrosion inhibition process. Thermodynamic parameters are extracted from Eqs. (5) and (6)^34^.

5 $$C_{R} = Ae^{{\frac{{ - E_{a} }}{RT}}} ,$$

6 $$C_{R} = \frac{RT}{{Nh}}\exp \left( {\frac{{\Delta S^{ * } }}{R}} \right)\exp \left( {\frac{{ - \Delta H^{*} }}{RT}} \right),$$

**Table 4:** Gravimetric parameters for Copper in 1.0 M HCl solution with and without AFM extract (250 mg L^–1^) at various temperatures.

Temperature (K)	AFM extract	*C*_*R*_ (μg cm^–2^ h^–1^)	*η*_*W*_ (%)
298	0	54.9	–
	+	4.9	91.0
313	0	57.3	–
	+	6.1	89.3
323	0	63.8	–
	+	11.3	82.2
333	0	75.2	–
	+	15.8	78.9

(*h* = 6.6261 10^−34^ m^2^ kg s^−1^, *R* = gas constant, *N* = 6.2022 1023 mol^−1^, and *A* = constant).

**Figure 4:**
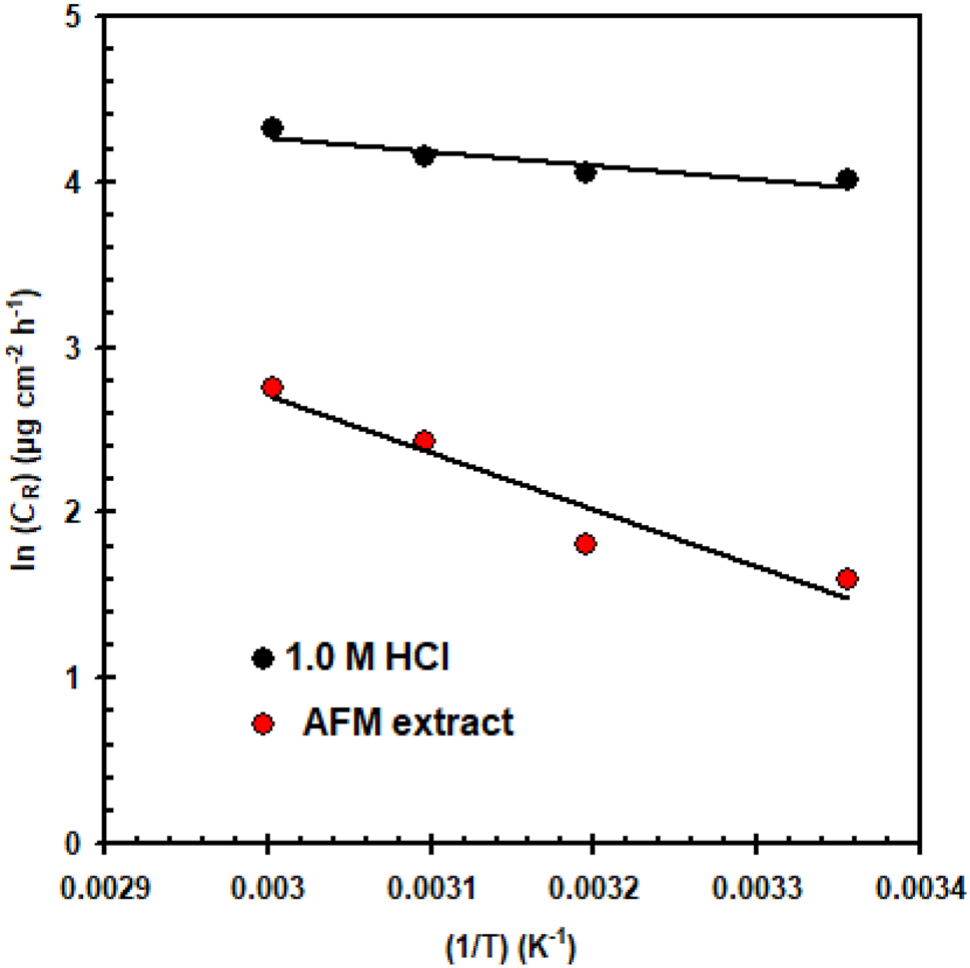
Arrhenius plot for copper in 1.0 M HCl solution in the presence/absence of AFM extract (250 mg L^–1^).

**Figure 5:**
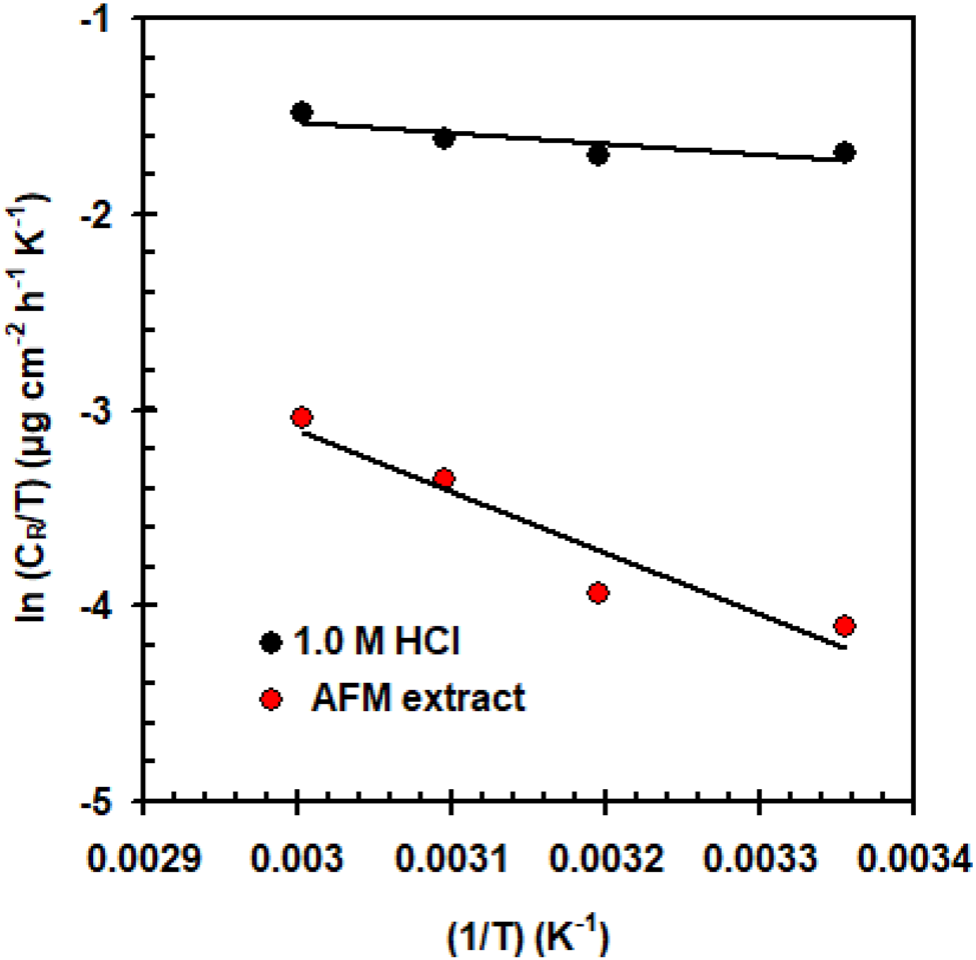
Transition state plot for copper in 1.0 M HCl solution in the presence/absence of AFM extract (250 mg L^–1^).

The *E*_a_ was determined using an Arrhenius diagram (Fig. 4) with or without of the 250 mg L^–1^ AFM extract.When AFM extract is added, the *E*_a_ increases to 28.6 kJ mol^–1^ from 7.15 kJ mol^–1^ (blank solution). Higher activation energy in the presence of AFM extract hinders down copper corrosion. The dimension of the double layer increases as a result of the adsorption of AFM extract on the surface of the copper, raising the energy barrier required to start the corrosion process. This was connected to the molecules of the AFM extract having favorable physical adsorption^35,36^. The values of ΔH* and ΔS* were obtained using the transition diagram (Fig. 5). When AFM extract is added, the ΔH* increases to 26.01 kJ mol^-1^ from 4.53 kJ mol^-1^ (blank solution). The positive magnitude of ΔH* indicates the endothermic process of copper oxidation in acidic solution^37,38^. From − 194 J mol^−1^ K^−1^ (blank solution) to − 174 J mol^−1^ K^−1^, there was a minor variation in the ΔS*. In addition, it is possible to attribute the shift in ΔS* from a negative value in the blank solution to a lower negative value in the AFM extract solution as a result of the rise in H_2_O entropy caused by H_2_O de-sorption over the copper surface in the presence of AFM extract^39^.

### Surface morphology studies

**Figure 6:**
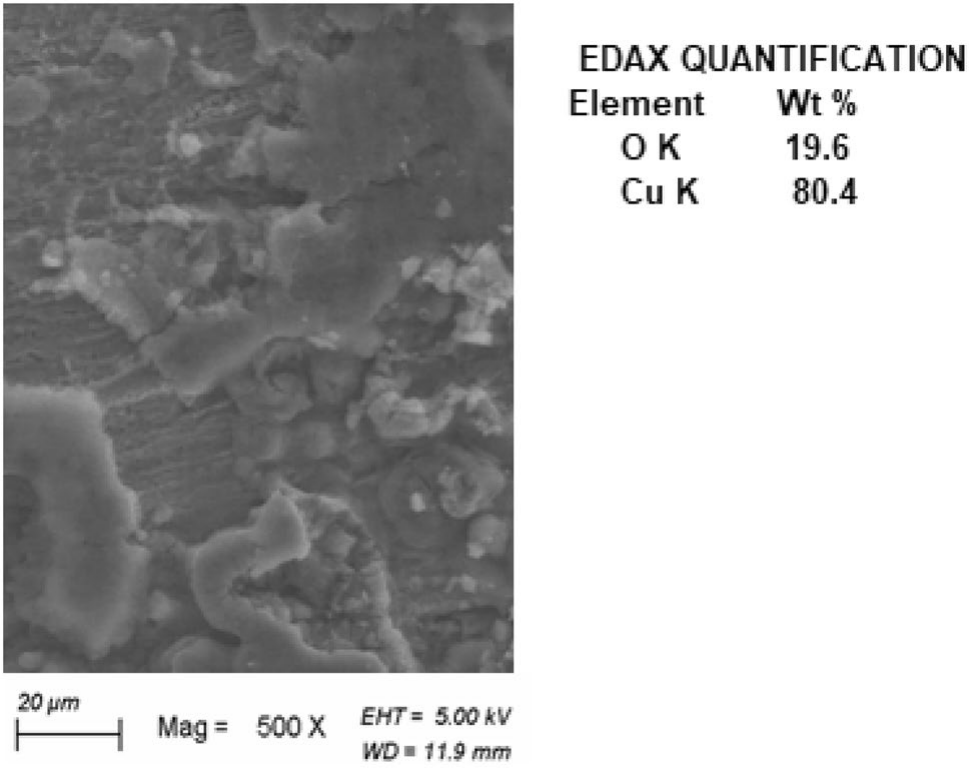
SEM and EDAX QUANTIFICATION for copper in 1.0 M HCl solution.

**Figure 7:**
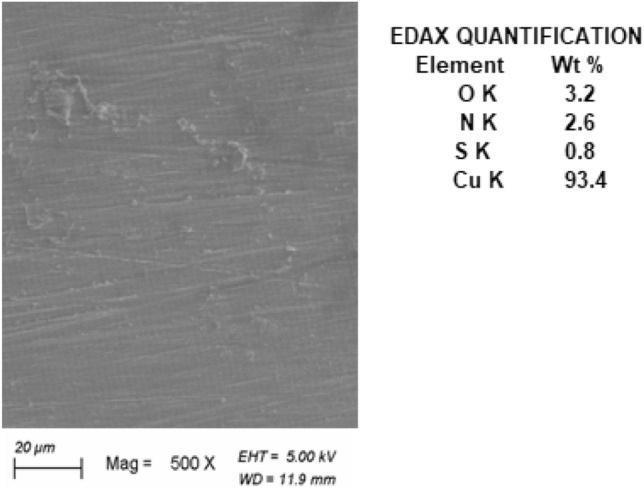
SEM and EDAX QUANTIFICATION for copper in 1.0 M HCl solution in the presence of AFM extract (250 mg L^–1^).

Figure 6 illustrates a SEM photo of Cu after 3 h immersion in 1.0 M HCl without AFM extract. The a view of the Cu surface in 1.0 M HCl (Fig. 6) indicates that the top layer of the metal has been severely corroded by copper dissolve when exposed to this aggressive solution, resulting in numerous cracks and pits evident on the copper’s surface. Cu-oxide is formed as a result of the chemical reaction of the copper's outer layer and the HCl solution. The corresponding EDAX includes O K (19.6%) and Cu K (80.4%), indicating the production of copper oxides CuO and/or cuprites Cu_2_O as products of corrosion on the copper's surface. whereas introducing 250 mg L^–1^ of AFM extract into the corrosive solution (Fig. 7) greatly decreased its corrosive properties and avoided corrosion of the copper surface, as evidenced through a significantly smooth copper surface being nearly totally coated by AFM extract molecules. The associated EDAX includes O K (3.2%), N K (2.6%), S K (0.8%), and Cu K (93.4%), indicating that the AFM extract molecules adsorb on the copper's surface.

**Figure 8:**
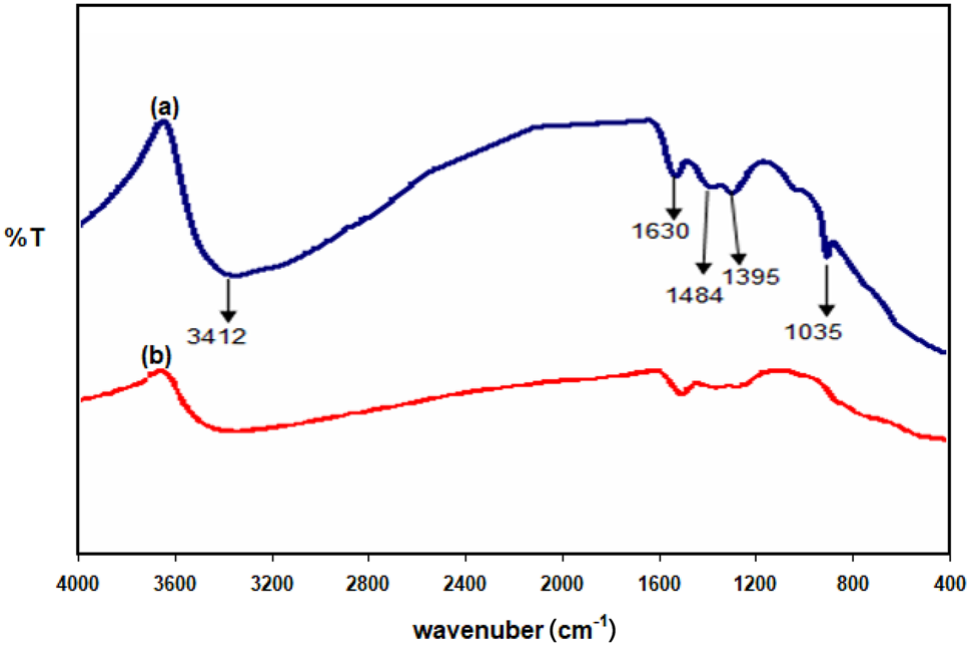
FTIR spectra of (a) AFM extract and (b) AFM extract adsorbed on copper in 1.0 M HCl solution in the presence of AFM extract (250 mg L^–1^).

The FTIR spectra (see Fig. 8a) derived from the pure AFM extract revealed peaks related to O–H (3412 cm^−1^), C–O stretching (1630 cm^−1^), benzene ring (1484 cm^−1^), C–N stretching (1232 cm^−1^), and C–O–C stretching (1035 cm^−1^). The results thereof revealed that the AFM extract contains nitrogen and oxygen in its functional groups, which is consistent with the chemical structures of common corrosion inhibitors. When comparing the AFM extract's spectrum to that of the solid corrosion result (Fig. 8b), shifts with regard to the benzene ring, O–H, C–N stretching, and C–O–C frequencies are seen. These findings pointed out that the surface of copper had absorbed AFM extract. The shifts in the spectra demonstrate that the functional groups in the components of the AFM extract interacted with copper during the contact.

## Conclusions

Aloe ferox Mill (AFM extract) can be utilized in the present research as a naturally generated corrosion inhibitor for copper in HCl solution. Tests using electrochemistry, SEM/EDAX/ FT-IR and gravimetric analysis were employed to assess the AFM extract's ability to suppress corrosion. AFM extract has an inhibitory ability that rises with concentration and reaches its maximum value (93.3%) at 250 mg L^–1^. The corrosion inhibition efficiency steadily drops with increasing temperature, pointing to a physisorption process.

Increasing the temperature has little effect on corrosion inhibition efficiency, suggesting that the AFM extract is an effective inhibitor at high temperatures. Copper corrosion gradually slows down when AFM extract is present because of the higher activation energy. In an acidic solution, the endothermic process of copper oxidation is shown by the positive magnitude of ΔH*.

## Data Availability

The datasets used and/or analysed during the current study available from the corresponding author on reasonable request.
